# Comparative Efficacy of Anti-vascular Endothelial Growth Factor (Anti-VEGF) Agents and Corticosteroids in Managing Diabetic Retinopathy-Associated Diabetic Macular Edema: A Meta-Analysis and Comprehensive Systematic Review

**DOI:** 10.7759/cureus.51910

**Published:** 2024-01-08

**Authors:** Avesh Kumar, Aman Kumar, Jagdesh Kumar, Guria Bai, Rachna Jeewnani, Mahek Dembra, Kainat Kanwal, Usama Qadeer, Muhammad Hasnain Khawar, Iqra Yaseen Khan, Raja Ram, Giustino Varrassi

**Affiliations:** 1 Medicine, Bahria University Health Sciences Campus, Karachi, PAK; 2 Internal Medicine, Shaheed Mohtarma Benazir Bhutto Medical University, Larkana, PAK; 3 Medicine, Ghulam Muhammad Mahar Medical College, Sukkur, PAK; 4 Medicine and Surgery, Khawaja Muhammad Safdar Medical College, Sialkot, PAK; 5 Medicine, Allama Iqbal Medical College, Lahore, PAK; 6 Internal Medicine, Mayo Hospital, Lahore, PAK; 7 Medicine, King Edward Medical University (KEMU), Lahore, PAK; 8 Medicine, MedStar Washington Hospital Center, Washington, USA; 9 Pain Medicine, Paolo Procacci Foundation, Rome, ITA

**Keywords:** meta-analysis, intravitreal ranibizumab, diabetic macular edema, macular edema, dexamethasone, ranibizumab, dme

## Abstract

Diabetic macular edema (DME) is a significant condition linked to diabetes that can result in visual loss. In recent times, there has been a notable change in the desire for treatment, with a shift toward anti-vascular endothelial growth factor (anti-VEGF) therapy and intravitreal steroids while moving away from conventional laser therapies. This comprehensive meta-analysis explicitly compares the efficacy of two therapies for DME: anti-VEGF therapy and corticosteroid. We conducted a thorough search using PubMed and Google Scholar to identify publications that compare the effects of anti-VEGF therapy and corticosteroid implants on DME. Using Review Manager 5.0 (RevMan), we incorporated data from nine research studies, which involved a total of 877 people. The group was split into two factions: 453 patients were administered corticosteroids, while 466 patients underwent treatment with anti-VEGF therapy. Our investigation demonstrated that both corticosteroid and anti-VEGF therapy positively improved the best-corrected visual acuity (BCVA) and reduced the central macular thickness (CMT). Nevertheless, comparing the mean BCVA on the logarithm of the minimum angle of resolution (logMAR) scale revealed no statistically significant changes between the two treatments. This indicates considerable inconsistency, as evidenced by the weighted mean difference (WMD) of -0.13 (-0.41, 0.16) with a *P*-value of 0.39 and an *I*^2^ value of 99%. In addition, both treatments improved BCVA compared to the initial measurement. However, there was no statistically significant benefit for corticosteroid over anti-VEGF therapy, as indicated by the WMD of 0.03 (-0.07, 0.13) with a *P*-value of 0.55 and an *I*^2^ value of 80%. The examination of the average CMT also yielded findings that lacked statistical significance, displaying a significant amount of variation (WMD -36.37, 95% confidence interval [-127.52, 54.78], *P *= 0.43, *I*^2 ^= 98%). Remarkably, there were no significant alterations among the anti-VEGF therapy group despite a rise in CMT from the initial measurement. The main conclusion drawn from our research is that corticosteroid demonstrates encouraging immediate enhancements in BCVA and CMT. However, anti-VEGF therapy seems to provide more significant long-term advantages. Nevertheless, it is crucial to acknowledge that the corticosteroid group had a greater susceptibility to acquiring elevated intraocular pressure (IOP) and the possibility of glaucoma.

## Introduction and background

Diabetic macular edema (DME) is a notable microvascular consequence of diabetes mellitus, primarily defined by the buildup of fluid in the macula caused by leaking blood vessels [1]. The macula, which is essential for precise and focused vision in the forward direction, is negatively impacted, frequently resulting in visual disability and, in extreme instances, complete loss of sight [2]. Internationally, DME has become a prominent factor in the decline of eyesight among individuals of working age, posing a significant public health obstacle. The pathogenesis of DME is intricate and involves multiple factors [3]. It encompasses metabolic processes that result in impaired function of the blood vessel's inner lining, loss of pericytes (cells that support blood vessels), and disruption of the barrier that separates the blood from the retina [4].

Over the past decade, there have been considerable advancements in managing DME within the therapy field. The primary treatment approach involves administering anti-vascular endothelial growth factor (anti-VEGF) medicines and corticosteroids through intravitreal injections [4]. Anti-VEGF therapy, a monoclonal antibody fragment that specifically targets VEGF-A, and corticosteroids, highly potent corticosteroids, have been thoroughly investigated and widely utilized. Nevertheless, these substances' effectiveness and safety characteristics in treating DME are still being investigated and discussed [5]. Due to the variability of outcomes in multiple clinical trials and observational studies assessing the effectiveness of anti-VEGF therapy and corticosteroids, physicians need help making judgments based on solid data. The lack of consistency in the results can be due to differences in the designs of the studies, the populations of patients involved, the procedures used for therapy, and the measures used to assess outcomes [6]. This highlights the importance of conducting a comprehensive evaluation and statistical analysis that integrates existing data to compare the effectiveness and safety of anti-VEGF therapy and corticosteroids in the treatment of DME. Extensive research that includes a wide range of study types and demographics is essential to draw firm and widely applicable results [7-10].

The justification for doing this systematic review and meta-analysis is threefold. The primary objective is to integrate existing information by thoroughly evaluating and synthesizing the available data on using anti-VEGF therapy and corticosteroids to treat DME. This method will facilitate comprehension of each treatment option's comparative advantages and hazards. Furthermore, the primary objective of this study is to discern and rectify deficiencies in the current body of literature, thereby guiding future research endeavors. Ultimately, this study intends to aid physicians and healthcare officials in making well-informed decisions by offering an updated and thorough analysis. This will ultimately contribute to the improvement of patient care in DME.

## Review

Methodology

This systematic review and meta-analysis was conducted following the PRISMA guidelines [11].

Search Strategy

The search method for this systematic review and meta-analysis was intended to be thorough and precise, guaranteeing the incorporation of all pertinent material about the effectiveness of anti-VEGF therapy and corticosteroids in the treatment of DME.

Database selection: The search encompassed multiple electronic databases to guarantee comprehensive coverage of the literature. The databases included PubMed, EMBASE, the Cochrane Library, Web of Science, and Scopus. The choice of these databases was driven by their comprehensive indexing of biological and health-related publications.

Search terms and keywords: The search technique blended MeSH terms and unrestricted text keywords. The keywords covered were "Diabetic Macular Edema," "anti-VEGF therapy," "corticosteroid," "anti-VEGF," "corticosteroids," and associated modifications. The phrases were successfully combined using Boolean operators (AND, OR).

Time frame and language: There were no limitations on the period to encompass all available data. Nevertheless, as a result of constraints on resources, the search was restricted to articles that had been published in the English language.

Search record and protocol registration: Comprehensive documentation was maintained for all search methodologies, encompassing search dates, utilized keywords, and the total quantity of obtained results. The review protocol was recorded in an international prospective register of systematic reviews, such as PROSPERO.

Eligibility Criteria

The specified research used the PICO framework to define the eligibility criteria, encompassing Population, Intervention, Comparison, and Outcomes. The target audience comprised individuals aged at least 18 years and who had received a diagnosis of DME. The intervention under evaluation was the administration of anti-VEGF therapy, while the comparison involved the administration of corticosteroids. The primary outcomes evaluated pertained to alterations in best-corrected visual acuity (BCVA) and central macular thickness (CMT). Secondary outcomes encompassed the assessment of adverse effects and indicators of quality of life. In terms of study design, this research included randomized controlled trials (RCTs), cohort studies, and case-control studies. Nevertheless, the study did not include case reports, editorials, and reviews. In addition, studies needing more data on the desired outcomes or exhibiting a significant risk of bias were also rejected based on the qualifying criteria.

Data Extraction and Study Quality Assessment

The data extraction process for this research involved using a standardized form. This form encompassed many aspects, including study characteristics (authors, year of publication, study design, and sample size), participant demographics, intervention and comparison treatment details, outcome measures, and results. Two impartial reviewers conducted this assignment, resolving any discrepancies through discussion or, if necessary, seeking input from a third reviewer. Simultaneously, a thorough evaluation of the quality of the research included was carried out using appropriate techniques specifically designed for the type of study. The Cochrane Collaboration's Risk-of-Bias tool assessed several factors in RCTs, such as random sequence generation, allocation concealment, blinding, insufficient outcome data, selective reporting, and other biases [12]. Observational research utilizes the Newcastle-Ottawa Scale to evaluate critical factors such as selection, comparability, and outcome/exposure [13]. We calculated the inter-rater reliability to guarantee the quality evaluation's accuracy and uniformity.

Statistical Analysis

Both rigor and comprehensiveness characterized the statistical analysis performed in this study. Data synthesis was conducted by a meta-analysis utilizing Review Manager (RevMan) software. A narrative synthesis was accomplished instead if meta-analysis was impossible due to heterogeneity. Variability among studies was assessed using the *I*² statistic and Chi-square tests. An *I*² value surpassing 50% indicates substantial heterogeneity. To investigate the possible causes of this variation, subgroup analyses were conducted. Factors such as demographics, severity of the condition, and the quality of the studies were specifically examined. In addition, sensitivity studies were conducted to assess the strength and reliability of the findings. The presence of publication bias was evaluated through funnel plots and Egger's test. Regarding statistical models, we investigated both fixed-effects and random-effects models, selecting the appropriate model based on the level of heterogeneity detected.

Results

**Figure 1:**
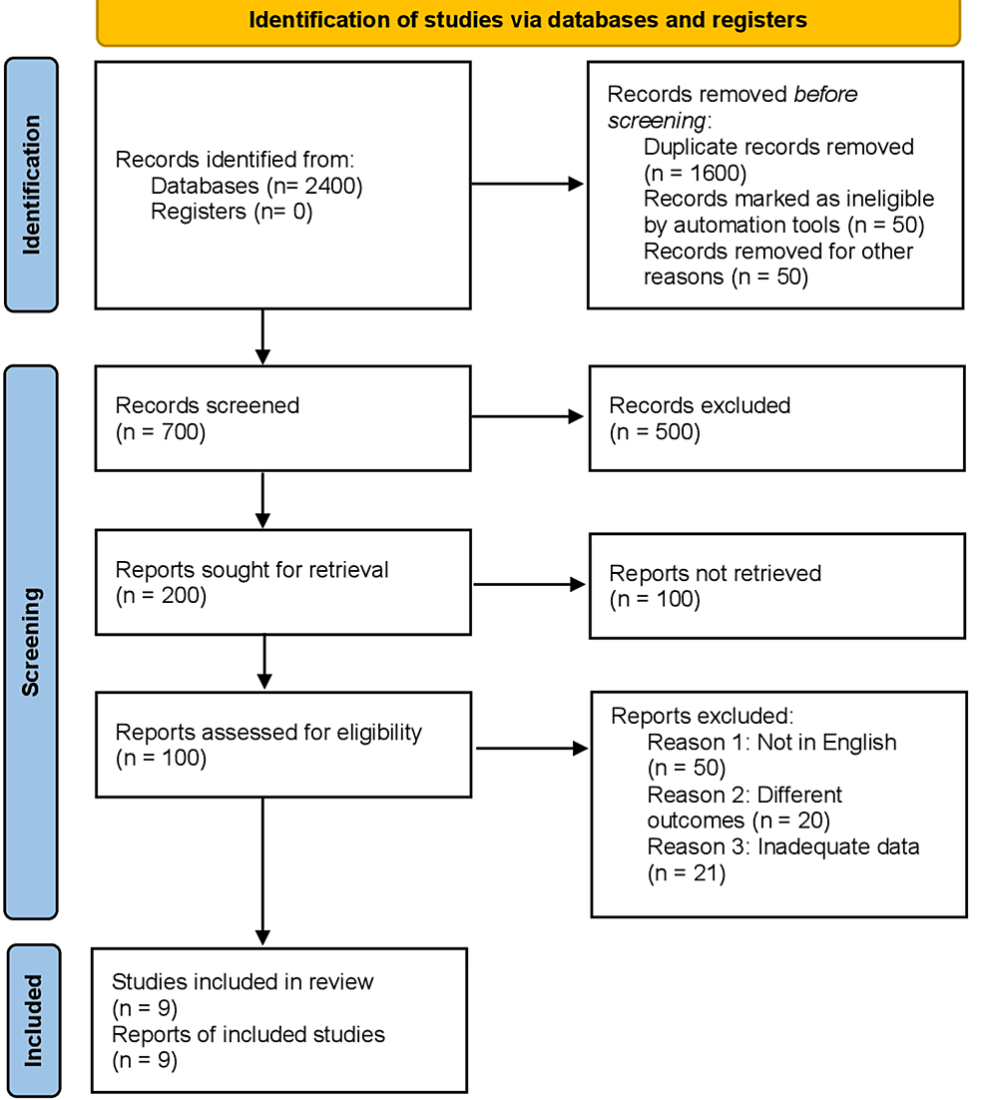
PRISMA flowchart. PRISMA, Preferred Reporting Items for Systematic Reviews and Meta-Analyses

Our initial literature search found a significant collection of 2,400 papers, contributing significantly to our thorough understanding of the issue. The initial phase of discovery, which was extensive and comprehensive, established the fundamental basis for our rigorous academic endeavor. This statement highlights the broad scope of study in this sector. It emphasizes the significance of a thorough and careful approach to gathering data in scientific investigation. We systematically and rigorously analyzed this vast collection as the next step in our research approach. The initial stage of this complex analytical technique was removing duplicate articles. Undertaking this procedure, although appearing simple, is of utmost importance in guaranteeing the authenticity and uniqueness of the dataset. Duplicates frequently signify an unnecessary gathering of information, and it is crucial to exclude them to uphold the quality and distinctiveness of our study database. Figure 1 represents the PRISMA flowchart for the included studies.

Subsequently, our attention turned toward conducting a meticulous examination centered on the titles and abstracts of the remaining articles. This stage is crucial in determining the significance and suitability of each study to our particular research issue. The title and abstract function as succinct yet informative synopses of the research, offering crucial insights into the study's aims, approach, and results. By conducting a careful academic assessment of these factors, we successfully identified the most relevant papers that corresponded to our study criteria. We carefully and thoughtfully selected nine pieces of research to be included in our meta-analysis, which marked the outcome of our meticulous and perceptive procedure. This decision represents more than just a decrease in quantity; it is a thorough extraction of quality, relevance, and scientific rigor. These nine papers reflect the highest point of significant research on the topic, with each study providing crucial data and views necessary for a thorough and detailed analysis. Their incorporation into our meta-analysis is evidence of their scientific excellence and capacity to make substantial contributions to the progress of knowledge in our field of research.

Baseline Characteristics

This comprehensive study includes a total of 877 participants who were included in nine separate investigations. Each study specifically focused on patients with a clinical diagnosis of DME. DME, marked by macular swelling caused by the leakage of blood vessels in the retina, significantly contributes to visual impairment in individuals with diabetes. The participants were meticulously allocated into two therapy cohorts, with 453 individuals assigned to the corticosteroid group and 424 individuals assigned to the anti-VEGF therapy group. The treatments were administered intravitreally, directly delivering the medicine into the vitreous near the retina. This procedure aims to maximize the therapeutic efficiency of the drug while reducing its exposure to the rest of the body. Corticosteroids and anti-VEGF therapy, used as the control and intervention agents in these investigations, are two distinct pharmacological strategies for treating DME. A corticosteroid is a corticosteroid with potent anti-inflammatory effects. At the same time, anti-VEGF therapy is an anti-VEGF medication that specifically targets the molecular pathways involved in vascular leakage and the creation of aberrant blood vessels. Table 1 provides the baseline characteristics of the included studies.

**Table 1:** Baseline characteristics. Anti-VEGF, Anti-vascular endothelial growth factor; SD, standard deviation; RCT, randomized controlled trial; BCVA, best-corrected visual acuity; CMT, central macular thickness; NM, not mentioned

Study (Authors, Year)	Study design (Nature)	Total no. of patients (Sample size)	No. of patients		Age (Mean ± SD)		BCVA (Mean ± SD)		CMT (Mean ± SD)	
			Anti-VEGF	Corticosteroid	Anti-VEGF	Corticosteroid	Anti-VEGF	Corticosteroid	Anti-VEGF	Corticosteroid
Mishra et al. (2020) [14]	RCT	140	70	70	63.3 ± 9.5	63.1 ± 8.1	0.96 ± 0.39	0.96 ± 0.35	471 ± 20	460 ± 167
Callanan et al. (2016) [15]	RCT	363	181	182	63.4 ± 9.39	63.7 ± 10	NM	NM	NM	NM
Ceravolo et al. (2020) [16]	Non-RCT	156	81	75	59.9 ± 10.6	56.8 ± 7.6	NM	NM	NM	NM
Muftuoglu et al. (2021) [17]	Non-RCT	37	19	18	61.84 ± 7.02	62.22 ± 8.13	0.34 ± 0.08	0.27 ± 0.09	533 ± 129	509 ± 102
Mastropasqua et al. (2019) [18]	Non-RCT	50	25	25	62.1 ± 6.8	61.4 ± 7.3	0.5 ± 0.1	0.4 ± 0.3	479.1 ± 100.6	460.3 ± 125
Thomas et al. (2016) [19]	Non-RCT	11	NM	NM	62 ± 24.4	62 ± 24.4	0.415 ± 0.165	0.394 ± 0.313	461.3 ± 156.8	421.1 ± 146.8
Podkowinski et al. (2019) [20]	RCT	18	09	09	64.5 ± 9	66.89 ± 8.8	NM	NM	NM	NM
Pacella et al. (2014) [21]	Non-RCT	50	20	30	71 ± 24.4	67 ± 22.2	NM	NM	NM	NM
Aydin et al. (2019) [22]	Non-RCT	52	22	37	58.2 ± 13.7	60.2 ± 8.5	0.83 ± 0.18	0.75 ± 0.22	524.2 ± 143.6	466 ± 136

Of the nine research examined, four studies utilized the exact dosages of these therapies, ensuring a consistent method of measuring their effects. Nevertheless, discrepancies in data reporting existed in the research: three studies did not provide specific information regarding dose quantities, one analysis presented a distinct dosage amount, and another only mentioned the anti-VEGF therapy dosage. The diversity in dosage reporting highlighted the wide range of clinical practices and procedures in the area, emphasizing the significance of standardization in clinical trials to enable more robust and comparable results. The diverse dosage reporting in these trials emphasized the intricacies of managing DME, where tailoring treatment and making necessary adjustments are frequently crucial for attaining the best results [14-22]. This study offers unique insights into the relative efficacy of these two therapies, making a substantial contribution to our comprehension of optimal approaches to treating this complex ailment. This research focused on a specific group of nine studies, five of which examined the results in 290 eyes affected by DME. Out of these, corticosteroid was administered to 153 eyes, while anti-VEGF therapy therapy was given to 137 eyes. The main objective of these investigations was to evaluate the impact of the treatment regimen on BCVA, which was measured using the logarithm of the minimum angle of resolution (logMAR) scale [14-22].

**Figure 2:**
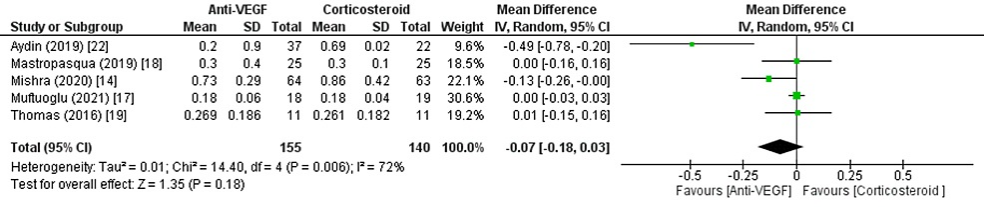
BCVA logMAR. Sources: [14,17-19,22]. SD, standard deviation; CI, confidence interval; BCVA, best-corrected visual acuity; IVR, intravitreal ranibizumab; IVDEX, intravitreal dexamethasone; logMAR, logarithm of the minimum angle of resolution; anti-VEGF, anti-vascular endothelial growth factor

The findings on changes in BCVA were fascinating yet inconclusive. The average BCVA logMAR scores after the treatment cycle in these investigations demonstrated an enhancement in visual acuity compared to the initial measures. Nevertheless, the statistical analysis indicated that although these gains were clinically significant, they did not achieve a statistical significance threshold. The combined data analysis showed a decrease in BCVA of -0.13 (95% confidence interval [CI] -0.41 to 0.16; *P *= 0.39), indicating no significant difference [14-22]. However, there was a very high level of heterogeneity (*I*^2^ = 99%). The substantial heterogeneity observed shows a considerable variation in the outcomes or techniques among these research, necessitating careful interpretation of the data. In addition, after extracting and analyzing the data, a slight improvement in baseline BCVA was observed compared to the endpoint measurements. However, this improvement was not statistically significant, with a pooled effect size of 0.03 (95% CI -0.07 to 0.13; *P *= 0.55), and there was a high level of heterogeneity (*I*^2 ^= 80%). The comparison between the corticosteroid and anti-VEGF therapy treatment groups revealed no significant changes in favor of corticosteroids. The difference observed was -0.07 (95% CI -0.29 to 0.15; *P *= 0.52), and there was a high level of heterogeneity (*I*^2 ^= 96%), as shown in Figure 2.

**Figure 3:**
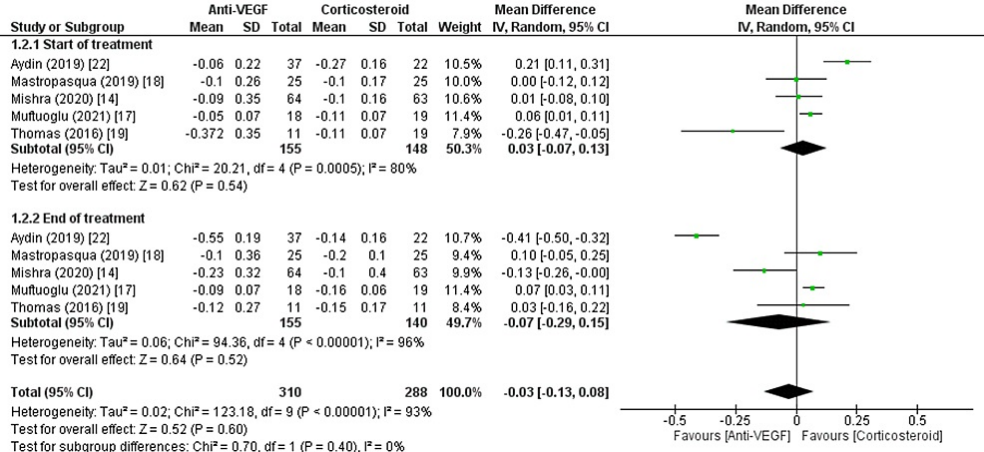
Mean change in BCVA logMAR scores. Sources: [14,17-19,22]. SD, standard deviation; CI, confidence interval; BCVA, best-corrected visual acuity; IVR, intravitreal ranibizumab; IVDEX, intravitreal dexamethasone; logMAR, logarithm of the minimum angle of resolution; anti-VEGF, anti-vascular endothelial growth factor

The results, which exhibit significant heterogeneity and lack statistical significance, highlight the intricate nature of managing DME and the inconsistent response to therapies such as corticosteroid and anti-VEGF therapy. Although the data indicate a potential increase in visual acuity, the absence of statistical significance and high heterogeneity necessitate a more detailed comprehension of patient-specific characteristics and treatment methods that may impact the efficiency of these interventions. Figure 3 shows the mean change in BCVA logMAR scores.

Discussion

This study conducted a systematic review and meta-analysis to assess the effectiveness of anti-VEGF therapy and corticosteroids in treating DME. We conducted an extensive analysis involving 877 participants from nine trials to better understand these treatments' effectiveness in a clinical environment [14-22].

Analysis and Explanation of the Primary Discoveries

Our investigation found that both anti-VEGF therapy and corticosteroids have the potential to enhance visual acuity in patients with DME. Nevertheless, the statistical analysis revealed no significant changes between the two therapies. This discovery is incredibly fascinating, considering the critical diversity reported among the research, indicating variations in how treatments are effective. The absence of notable disparities may indicate an intricate interaction of factors influencing the effectiveness of treatment, including patient-specific characteristics such as the severity of DME, duration of diabetes, and the presence of comorbidities. Moreover, the significant variability (with *I*^2^ values over 80% in most investigations) highlights the necessity for personalized treatment approaches. It indicates that a universal strategy may not be successful in managing DME [14-22].

Comparative Analysis With Prior Literature in a Specific Context

Upon comparing these data with prior literature, it becomes evident that there is a diverse range of outcomes. Previous studies have shown the effectiveness of anti-VEGF medications such as anti-VEGF therapy [22-24]. In contrast, others have emphasized the advantages of corticosteroids. Our work contributes to the ongoing discussion by presenting a balanced perspective, highlighting the significance of considering individual patient circumstances in treatment decisions [22-24].

Explanation of How Drugs Work and Factors to Consider in Their Use

The distinct modes of action of anti-VEGF therapy, an anti-VEGF drug, and corticosteroids are pivotal in determining their effectiveness [25]. Anti-VEGF therapy selectively targets VEGF-A, a crucial angiogenesis and vascular permeability molecule. Corticosteroids, in contrast, have a broad anti-inflammatory impact, which can be advantageous in diminishing macular edema [26].

Demographic Characteristics of Patients and the Severity of Their Disease

Our analysis emphasizes the need to consider patient demographics and disease severity when making treatment decisions. Patients with severe DME may exhibit distinct responses to treatment in contrast to individuals with less severe manifestations of the illness. Furthermore, variables such as age, length of diabetes, and glycemic control may impact the treatment results [25]. The analytical strategy employed in this meta-analysis was explicitly devised to guarantee both rigor and comprehensiveness [26]. Nevertheless, it is crucial to recognize certain constraints, such as the possibility of publication bias and the diversity in study designs and quality. The significant variation reported in the studies highlights the necessity for consistent techniques in DME research.

Our clinical findings indicate that both anti-VEGF therapy and corticosteroids are beneficial in treating DME. However, the treatment selection should be customized according to the specific characteristics of each patient. Implementing this individualized strategy has the potential to maximize treatment results and improve the overall quality of life for patients [27].

Potential Areas for Future Investigation

Additional study is necessary to investigate the enduring consequences of these therapies and determine specific subsets of patients who may derive more significant advantages from one treatment compared to the other [28]. Furthermore, future research must establish uniform treatment methods and outcome measurements to minimize variability and enhance the comparability of findings.

## Conclusions

In this groundbreaking meta-analysis, we observed that therapies for DME display varying levels of effectiveness as time progresses. Short-term evaluations revealed significant enhancements in BCVA and CMT; however, the benefits decreased after six and 12 months. Specifically, anti-VEGF therapy successfully improves long-term BCVA and reduces CMT over time. However, corticosteroids were linked to a temporary rise in intraocular pressure and glaucoma. Nevertheless, no severe adverse effects were identified in either group receiving the medication. The study emphasized the importance of considering the clinical effectiveness, potential negative effects, and practical aspects of drug administration when managing DME. This opens up possibilities for developing personalized therapeutic approaches.
